# Development of Point-of-Care Biosensors for COVID-19

**DOI:** 10.3389/fchem.2020.00517

**Published:** 2020-05-27

**Authors:** Jane Ru Choi

**Affiliations:** ^1^Centre for Blood Research, Life Sciences Centre, University of British Columbia, Vancouver, BC, Canada; ^2^Department of Mechanical Engineering, University of British Columbia, Vancouver, BC, Canada

**Keywords:** POC biosensors, PDMS, paper, flexible materials, COVID-19

## Abstract

Coronavirus disease 2019 (COVID-19) outbreak has become a global pandemic. The deleterious effects of coronavirus have prompted the development of diagnostic tools to manage the spread of disease. While conventional technologies such as quantitative real time polymerase chain reaction (qRT-PCR) have been broadly used to detect COVID-19, they are time-consuming, labor-intensive and are unavailable in remote settings. Point-of-care (POC) biosensors, including chip-based and paper-based biosensors are typically low-cost and user-friendly, which offer tremendous potential for rapid medical diagnosis. This mini review article discusses the recent advances in POC biosensors for COVID-19. First, the development of POC biosensors which are made of polydimethylsiloxane (PDMS), papers, and other flexible materials such as textile, film, and carbon nanosheets are reviewed. The advantages of each biosensors along with the commercially available COVID-19 biosensors are highlighted. Lastly, the existing challenges and future perspectives of developing robust POC biosensors to rapidly identify and manage the spread of COVID-19 are briefly discussed.

## Introduction

Coronavirus disease 2019 (COVID-19) is an infectious illness caused by severe acute respiratory syndrome coronavirus 2 (SARS-CoV-2) (Chen L. et al., 2020; Hu et al., 2020). On 20 January 2020, World Health Organization (WHO) declared the outbreak of COVID-19 a global public health emergency of international concern (Zheng et al., 2020). The incidence of COVID-19 has increased drastically, with more than three million cases reported worldwide, causing more than 200,000 deaths (Baud et al., 2020). The clinical manifestation of COVID-19 ranges from mild illnesses such as fever, cough and dyspnea to life-threatening syndromes, including pneumonia, acute respiratory distress syndrome, or even death (Bedford et al., 2020; Bernheim et al., 2020). COVID-19 has high transmission capability, making the prevention and control difficult (Chen S. et al., 2020). As there is no specific antiviral treatment or vaccine for COVID-19, early and prompt diagnosis is important to reduce the risk of life-threatening complications and mortality through appropriate health care (Lee et al., 2020; Xiao and Torok, 2020).

With the advances in POC testing, chip-based [e.g., polydimethylsiloxane (PDMS) biosensors] and paper-based biosensors [e.g., lateral flow test strips or three-dimensional (3D) paper-based microfluidic biosensors] have been developed for rapid diagnosis of infectious diseases (Choi et al., 2017; Yew et al., 2018; Zhang et al., 2019). They are widely used to detect antibodies, antigens or nucleic acids in crude samples such as saliva, sputum, and blood based upon colorimetric, fluorescent, or electrochemical detection approaches (Choi et al., 2015; Tang et al., 2017a; Yee et al., 2018). They offer many advantages such as being Affordable, Sensitive, Specific, User-friendly, Rapid and Robust, Equipment-free, and Deliverable to end users (ASSURED) (Gong et al., 2017; Tomás et al., 2019). The result can be obtained in a fast and simple manner, which allows rapid decision-making, hence minimizes the risk of human-to-human transmission.

In view of the escalating demand for rapid diagnosis of COVID-19, a mini review that summarizes the recent progress in developing POC biosensors for COVID-19 is highly desirable. In this review article, the most recent advances in POC biosensors, including both chip-based or paper-based biosensors for the detection of COVID-19 infection are reviewed. The advantages of each biosensors along with the commercially available COVID-19 biosensors are summarized. Finally, the existing challenges and future perspectives of developing robust and fully integrated POC biosensors for COVID-19 are briefly discussed.

## Development of Point-of-Care Biosensors for COVID-19

**Figure 1:**
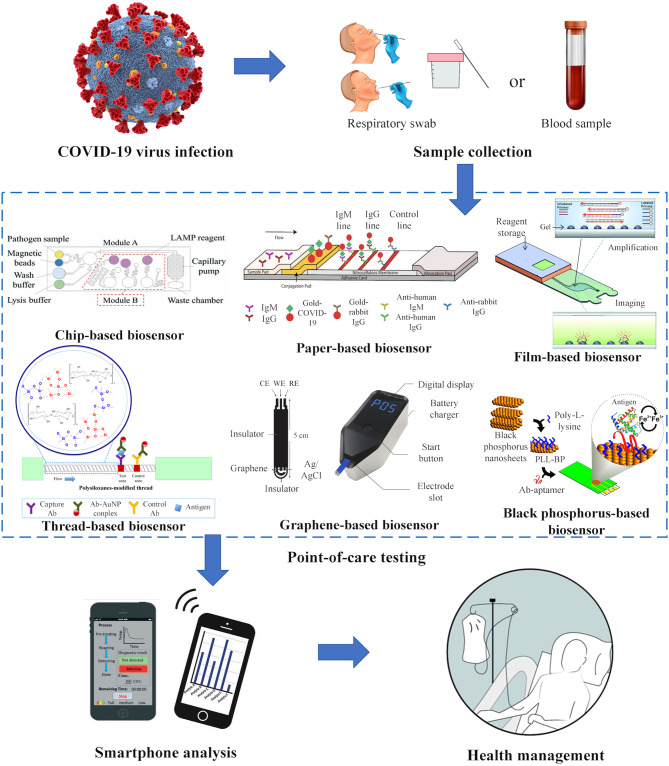
Point-of-care biosensors for COVID-19. Respiratory and blood samples are collected for the detection of viral nucleic acids and human antibodies against the virus. Point-of-care biosensors such as chip-based biosensors (Ma et al., 2019), paper-based biosensors (Li et al., 2020), film-based biosensors (Kukhtin et al., 2019), thread-based biosensors (Choi et al., 2018a), graphene-based biosensors (Kampeera et al., 2019), and black phosphorus-based biosensors (Kumar et al., 2016) offer tremendous potential for identifying and managing the spread of COVID-19. Rapid onsite analysis can be performed using a smartphone for appropriate health management.

In general, there are two types of rapid POC tests that can detect COVID-19 infections, which are nucleic acid and antibody (Ab) tests (Sheridan, 2020). The nucleic acid test is usually performed by detecting the presence of virus in patient's sputum (or saliva) or nasal secretions (snot) (Zhifeng et al., 2020). Such test is good at detecting the virus at early stage of infection or even before the symptoms appear. On the other hand, the antibody test strip (IgG/IgM test) is performed by collecting patient blood samples that contain antibodies against the virus (Li et al., 2020). In general, about 5 days after initial infection, the virus triggers the immune response which stimulates the production of both IgM and IgG in blood that fight against the virus (Thevarajan et al., 2020). These antibodies can be detected in patient plasma, serum or whole blood. The existing POC biosensors and commercial products for COVID-19 are summarized in Table 1. In fact, compared to the existing POC biosensors, quantitative real-time polymerase chain reaction (qRT-PCR), the gold standard for COVID-19, shows a higher clinical sensitivity and specificity, which are 79–96.7 and 100%, respectively (He et al., 2020). The clinical sensitivity and specificity of commercial POC biosensor (i.e., IgG/IgM lateral flow test strip) are 86.43–93.75 and 90.63–100%, respectively. The POC biosensors which are potentially used for COVID-19 are sample-to-answer chip-based biosensors, paper-based biosensors or other material-based biosensors (Figure 1) which are briefly discussed in the following sections.

**Table 1:** Point-of-care biosensors and commercial products for COVID-19.

	**Commercial product for COVID-19**	**Sample volume (μL)**	**Limit of detection (LOD)**	**Clinical sensitivity (%)**	**Clinical specificity (%)**	**Advantages**	**Limitations**
Chip-based biosensor (Loo et al., 2017; Yin et al., 2020)	–	10	30–1,000 CFU/mL	–	–	Low sample volume Allows on-chip sample-to-answer nucleic acid testing	Complex fabrication process Requires skilled personnel Clean room is usually required for fabrication
Paper-based biosensor (Choi et al., 2016a; Tang et al., 2017c)	2019-nCoV IgG/IgM detection kit (Biolidics)	20	–	91.54	97.02	Simple fabrication and operation processes User-friendly Cost-effective Lateral flow test strip allows high-scale production which remains the most popular screening option	Lack of quantification
	Clungene COVID-19 IgM/IgG rapid test cassette (Hangzhou Clongene Biotech)	10	–	87.01	98.89		
	COVID-19 IgG/IgM rapid test (Aytu BioScience)	20	–	91.9	100		
	COVID-19 IgG/IgM rapid test device (RayBiotech)	25	–	90.44	98.31		
	COVID-19 IgM-IgG rapid test (BioMedomics)	20	–	88.66	90.63		
	qSARS-CoV-2 IgG/IgM rapid test (Cellex)	10	–	93.75	96.4		
	One step COVID-19 IgM/IgG antibody test kit (Artron Laboratories Inc.)	10	–	93.4	97.7		
	Rapid response COVID-19 IgG/IgM rapid test (BTNX)	10	–	90.0	98.7		
	SGTi-flex COVID-19 IgM/IgG (Sugentech)	10	–	91	96.67		
	Wondfo SARS-CoV-2 antibody test (Guangzhou Wondfo Biotech)	10	–	86.43	99.57		
	Standard Q COVID-19 IgM/IgG Duo (SD Biosensor)	10	–	90.6	96.1		
	COVID-19 IgM/IgG rapid test kit (Aurora)	10	–	90.21	96.2		
	COVISURE COVID-19 IgM/IgG rapid test (Cardinal Health)	20	–	93.5	100		
Thread-based biosensor (Choi et al., 2018a)	–	20	5,000 CFU/mL	–	–	Much simpler fabrication than that of lateral flow test strip User-friendly Cost-effective	Lack of quantification
Film-based biosensor (Kukhtin et al., 2019)	–	500	30 CFU/mL	–	–	Low cost alternative to glass High polymerase chain reaction (PCR) compatibility of the film substrate Low background fluorescence Transparency of film allows optical inspection Able to withstand thermal cycling	Complex fabrication process
2D material-based biosensor (Kumar et al., 2016; Kampeera et al., 2019)	–	5	~0.01 CFU/mL cell or 0.5 pg/mL protein	–	–	High sensitivity and selectivity due to excellent electrochemical properties of 2D materials	Requires nanomaterial synthesis prior to biosensor fabrication Lengthy fabrication process (1-2 days)

### Chip-Based Biosensors

Chip-based biosensors have been broadly used for point-of-care diagnosis of infectious diseases. They are mainly made of PDMS or poly(methyl methacrylate) (PMMA) (Zarei, 2017a; Zhang et al., 2017). These biosensors allow automated, precise manipulation of fluid flow with small volume of samples. They are able to minimize reagent consumption which enables high throughput analysis (Dincer et al., 2017; Nasseri et al., 2018). PDMS chip-based biosensors, in particular, have attracted scientific interest due to its biocompatibility, high transparency and cost-effectiveness (Nayak et al., 2017). They have been explored in both antibodies and nucleic acid detection for monitoring infectious diseases (Wang et al., 2016; Darwish et al., 2018). Recent studies have focused on the development of sample-to-answer platform for nucleic acid testing as they are more sensitive and specific than antibody assays. For example, a study has developed a sample-to-answer “lab-on-a-disc” biosensor (Loo et al., 2017). This PDMS-based biosensor consists of multiple channels that allows automated nucleic acid extraction, isothermal amplification [loop-mediated isothermal amplification (LAMP)] and real time signal detection. SYTO-9 dye is used for detection, which binds to the double-stranded DNA from LAMP reaction and emits green fluorescence. The intensity of fluorescence signal indicates the amount of target detected. The biosensor is self-contained for performing sample heating and chemical lysis for both extraction and amplification processes. The entire sample-to-answer processes take ~2 h. While the platform is portable, the size of biosensor is relatively large, which requires further improvement. The biosensor can be readily customized to detect COVID-19 viruses in the near future.

More recently, one group has integrated digital amplification process into a sample-to-answer chip-based biosensor to quantify nucleic acids which further simplifies the entire process of nucleic acid testing (Yin et al., 2020). The biosensor integrates nucleic acid extraction, multiplex digital recombinase polymerase amplification (RPA) and fluorescence detection into a single biosensor. It is mainly made of PDMS layers with a glass slide as a supporting substrate. The specific primer mixture and all of the reaction solution for RPA except magnesium acetate are added onto three different detection areas and lyophilized. Once the sample is added, the cell is lysed and the nucleic acids bind to the magnetic beads. Following the washing and elution steps, the reagents are passively driven into the digital RPA area using vacuum-based self-priming approaches for isothermal amplification, followed by fluorescent imaging. As the fluorescence probe is labeled with carboxyfluorescein (FAM) which is detectable by the UV light, the fluorescence signal intensity is proportional to the concentration of amplicons. The biosensor could achieve three main steps of nucleic acid testing within 45 min without requiring complex instrument and control systems. This integrated biosensor shows immense potential to rapidly and accurately detect COVID-19 in patient samples.

Besides fluorescence signal detection, colorimetric detection approach has also been used in chip-based biosensors. The output of isothermal amplification (e.g., LAMP) has been visualized by colorimetric indicators [e.g., calcein or hydroxynaphthol blue (HNB)] that interact with amplicons or byproducts (i.e., pyrophosphate; Seok et al., 2017; Quyen et al., 2019; Wang et al., 2019). Calcein binds manganese ions that quench fluorescence and the complex interacts with pyrophosphate to express fluorescent signal (Dou et al., 2019). HNB changes color from violet to sky blue upon reacting with pyrophosphate (Yang et al., 2018). To improve the portability and functionality of chip-based biosensors, one has developed an automated, portable sample-to-answer biosensor coupled with a smartphone for colorimetric detection of pathogens (Ma et al., 2019). It consists of a microfluidic structure layer, a hydrophilic layer, a PDMS hydrophobic layer and a glass substrate. The biosensor is able to (i) purify pathogens with specific affinity reagent pre-conjugated to magnetic beads, (ii) conduct lysis at low temperatures, (iii) perform LAMP, and (iv) quantify the results based on colorimetric signals. HNB is used as an indicator or visual dye for amplicons. As mentioned, the LAMP reaction changes the color of LAMP products from violet to sky blue. The entire process is about 40 min which is automatically performed and being monitored using a smartphone. Future work should include storing the reagents on chip to make it more applicable at remote settings.

### Paper-Based Biosensors

Paper-based biosensors have attracted more significant attention for use in POC testing as compared to chip-based biosensors owing to their cost-effectiveness, biodegradability as well as ease-of-fabrication, functionalization and modification (Hu et al., 2017; Böhm et al., 2018; Choi et al., 2019). With these characteristics, they are able to achieve rapid, onsite POC testing in remote settings (Tang et al., 2017b). Lateral flow test strips, in particular, have been widely used for the detection of COVID-19. They are designed to detect IgG and IgM in patient whole blood, serum and plasma samples (Li et al., 2020; Sheridan, 2020). Each test strip generally consists of (i) a sample pad to add patient samples, (ii) a conjugate pad containing COVID-19 antigen conjugated with gold nanoparticles (gold-COVID-19) and gold-rabbit IgG, (iii) a nitrocellulose membrane that consists of a control line coated with goat anti-rabbit IgG, an IgG test line coated with anti-human IgG, an IgM test line coated with anti-human IgM as well as (iv) an absorbent pad that absorbs waste (Li et al., 2020; Sheridan, 2020). In the presence of IgM and/or IgG in patient samples, the antibodies react with gold-COVID-19 antigen to form a complex, which moves across the nitrocellulose membrane and interact with the anti-IgM and/or IgG at their respective test line. The gold-rabbit IgG in turn reacts with anti-rabbit IgG coated at the control line to produce a visible red color. A positive IgM and a negative IgG or positive at both lines indicate a primary or acute infection, while a positive IgG with a negative IgM shows a secondary or later stage of infection (Du et al., 2020; Li et al., 2020).

Apart from antibody testing, some lateral flow test strips with sample-to-answer capability have been used for nucleic acid testing which could potentially detect COVID-19 nucleic acids in respiratory samples. For instance, a group has introduced a fully integrated paper-based biosensor that involves three main steps of nucleic acid testing (i.e., nucleic acid extraction, LAMP, and detection), producing colorimetric signal detected by lateral flow test strip (Rodriguez et al., 2016). However, this integrated biosensor requires an external heat block for amplification. To simplify the platform for POC applications, a small and portable heater has been developed in combination with four-layered paper-based sample-to-answer biosensor (Choi et al., 2016a). This biosensor consists of Fast Technology Analysis (FTA) card and glass fiber for nucleic acid extraction and LAMP, along with an integrated lateral flow test strip for visual detection. Each functional layer is separated by hydrophobic polyvinyl chloride substrates that control the fluid flow from one layer to another. To further simplify the processes, a semi-automated, fully disposable and integrated paper-based biosensor has been developed (Tang et al., 2017c). This integrated biosensor consists of a paper-based valve and a sponge-based reservoir to extract nucleic acids from crude samples, a portable battery and a heater integrated into the platform for isothermal amplification (i.e., helicase dependent amplification, HDA) as well as a lateral flow test strip for colorimetric detection. The proposed biosensor allows on board reagent storage with the use of sponges and equipment-free isothermal amplification which significantly simplifies user steps. More recently, lateral flow test strip has been combined with paper folding technologies for sample preparation, LAMP and lateral flow detection (Reboud et al., 2019). The integrated test strip consists of buffer chambers that regulate fluid flow, acetate films which prevent sample evaporation, filter paper-based valves that prevent LAMP reagent from mixing with other reagents, and a lateral flow test strip. This integrated technology is suitable for use in resource-limited settings to test crude samples (e.g., sputum), offering great potential to rapidly detect nucleic acids of COVID-19 (<50 min).

Besides lateral flow test strips, several 3D paper-based microfluidic biosensors have been developed to detect proteins or nucleic acids at the POC (Xia et al., 2016; Lam et al., 2017). These biosensors usually detect targets based on fluorescence or colorimetric detection approaches. Metal ions (e.g., magnesium, calcium, or silver) have been used in 3D paper-based microfluidic biosensors to react with base (purine or pyrimidine) from double stranded DNA to form a stable complex that show fluorescent signal upon UV irradiation. For example, a fully integrated and foldable biosensor encapsulated with agarose have been developed for long-term reagent storage and multiplex fluorescence detection (Trinh et al., 2019). The biosensor consists of a reaction zone and a detection zone. Agarose that carries LAMP reagents and silver nitrate (reaction indicator) is deposited and stored in reaction and detection zones for at least 45 days. The sample is added into reaction zones which are then closed with an adhesive sealing film to avoid sample evaporation before being placed on a portable heater for amplification. Following the amplification process, the sealing film is removed and the detection zone is folded and soaked into the reaction zone. UV light is used to visualize the reaction between amplicons and silver ions. The brown color intensity of the test zone increased along with the increased concentration of amplicons. The biosensor is simple and user friendly, which is expected to detect COVID-19 nucleic acids in patient samples.

More recently, as a new alternative, fuchsin has been explored for the detection of DNA amplicons based upon colorimetric detection approach using a 3D paper-based microfluidic biosensor (Trinh and Lee, 2019). In the absence of DNA amplicons, addition of sodium sulfite molecule and fushsin produces fushsin leucosulfonic acid or leucofushsin which is colorless. In the presence of DNA amplicons, aldehyde groups of the DNA bind to sulfonate groups and the bond between hydrogen sulfite and the central C atom is broken, producing the fuchsin with chromophoric structure, which appears to be purple signals (Mello and de Campos Vidal, 2017). The proposed biosensor is composed of a sample zone, a reaction zone and a detection zone. The detection zone consists of paper strips with fuchsin stained lines. Briefly, sample is injected into the sample zone which is sealed with an adhesive sealing film to prevent sample evaporation. The reaction zone is folded to bind to the sample zone and the biosensor is turned upside down to enable the flow of sample from sample zone to reaction zone. The biosensor is heated on a hot plate at 65°C for 30 min for LAMP. After LAMP, the sealing film is peeled off and the hydrochloric acid is injected into the reaction zone. Sodium sulfite is subsequently added and the changes of fuchsin-stained line color is observed. Unlike the above-mentioned biosensors, this biosensor produces simple colorimetric signals detectable by the naked eyes without requiring any external readers, which shows promising for rapid diagnosis of COVID-19 infections at the POC.

### Other Biosensors

Apart from developing chip-based and paper-based biosensors, other material-based biosensors such as textile-based, film-based or carbon-based biosensors have also been introduced for potential use for COVID-19 (Parrilla et al., 2016; Afsahi et al., 2018; Mogha et al., 2018). They are developed to improve the functionality and detection sensitivity of the existing biosensors, providing more promising choices for practical use. Textile-based biosensors such as thread-based, fabric-based or cloth-based biosensors have been developed with simple fabrication processes and improved assay performance. One study has incorporated polysiloxanes with tunable hydrophobicity into thread-based biosensors to delay fluid flow in lateral flow assay and improve detection sensitivity (Choi et al., 2018a). Similar to conventional lateral flow test strip, the fluidic delay in thread increases interactions between gold nanoparticles-antibodies (AuNP-Ab) and targets. With the optimum conditions, the increase in interactions produces more AuNP-Ab-target complexes, showing 10-fold more sensitive than the unmodified biosensors. This biosensor is simple-to-fabricate and highly sensitive, which shows immense potential in detecting IgG and IgM in COVID-19 patients for appropriate health monitoring.

In addition to textile-based biosensors, film-based biosensors have been developed to detect infectious microorganism from crude samples. For instance, a film-based biosensor has been introduced which consists of a transparent polyester film substrate, a sample chamber, a cover, a reaction chamber and a waste chamber (Kukhtin et al., 2019). Microarray 2-(hydroxyethyl)methacrylamide gel elements are synthesized with the incorporation of oligonucleotides into gels and polymerization under UV irradiation. The gel elements are covalently attached to the substrate. Briefly, both sample and master mix are first introduced into chambers. The capillary action promotes uniform filling of the chamber and amplification is subsequently performed. Wash buffer is then introduced and the pressure leads the waste to the waste chambers. Lastly, fluorescent imaging and analysis are performed. The flexible film substrate used in the study offers several advantages for POC testing: (i) allows covalent attachment of gel without pre-treatment processes, (ii) has low background fluorescence, (iii) has transparent properties that allows optical inspection, (iv) able to withstand thermal cycling and compatible to amplification processes. This flexible film allows easy fabrication and is user-friendly, which costs ~500 times less than other substrates (i.e., glass), demonstrating its potential for detecting COVID-19 virus in patient samples.

Apart from these, 2D materials such as graphene or black phosphorus have also been integrated into biosensors for POC diagnosis which could potentially be used for COVID-19 testing (Jin et al., 2017; Choi et al., 2018b; Liu et al., 2018). For example, a recent study has introduced a portable graphene-based electrochemical biosensor for highly sensitive POC testing (Kampeera et al., 2019). Graphene-based electrodes are constructed by screen-printing mainly due to low cost, ease of fabrication and high production rate. The screen-printed graphene electrodes (SPGE) are synthesized by either substituting carbon with graphene or incorporating graphene into carbon paste. SPGE have been reported to show superior electrochemical properties than commonly used screen-printed carbon electrodes (SPCE) by having a higher electron transfer rate and a larger electrochemical surface area. After nucleic acid extraction, LAMP is performed using a simple heating block. The interaction between SPGE and amplicons results in a shift in cathodic current which stems from the intercalation of redox probe to double-stranded DNA. This phenomenon enables quantification of LAMP product. A portable mini potentiostat is used in combination with SPGE for on-site detection. The next step should be the integration of extraction platform into the proposed biosensor to make it more portable and user-friendly.

Besides graphene, black phosphorus (BP) or phosphorene-based biosensors have also been extensively explored for medical diagnosis (Qian et al., 2017; Ge et al., 2019; Luo et al., 2019). BP displays excellent electrochemical properties which enhances assay sensitivity and selectivity owing to its inherent redox properties. For example, a recent study has reported the development of a label-free electrochemical biosensor with an aptamer-functionalized BP nanostructured electrode (Kumar et al., 2016). The BP nanosheets are coated with poly-L-lysine (PLL) that allows functionalization of BP with antiAb-aptamers. The aptamers are immobilized onto the nanosheets via the coulomb interaction between aptamers and PLL. The presence of target antigens causes the direct oxidization of iron (ii) to iron (iii) at the electrode surface through the mechanism of electron transfer. As compared to reduced graphene oxide (rGO), BP-based biosensors show a higher detection sensitivity and specificity, achieving the detection limit down to pg level compared to ng level achieved by rGO biosensors. The proposed biosensor would allow highly sensitive detection of IgG or IgM against coronavirus in patient blood samples.

## Conclusion and Future Perspectives

In summary, this review article discusses the POC biosensors that made of PDMS, paper and other flexible materials such as textile, film, and carbon nanosheets for rapid diagnosis of COVID-19. The cost-effectiveness, simplicity, rapidity and portability of these biosensors play a crucial role in POC applications. Antibody tests are suitable to detect the late stage of infections while nucleic acid tests detect the presence of nucleic acids (viruses) at the early stage of infection, showing a higher sensitivity and specificity than antibody tests. However, current nucleic acid tests require three key steps (i.e., nucleic acid extraction, amplification, and detection), involving more complicated processes than that of antibody tests. In fact, most of the commercial POC biosensors for COVID-19 are paper-based biosensors or lateral flow test strips for antibody detection (IgG and IgM) that produce colorimetric signal. While these antibody tests display lower specificity compared to nucleic acid tests, they have helped shortening the turnaround time for rapid diagnosis, allowing fast decision making. Future work should include specificity improvement or combination with other tests such as rapid nucleic acid tests to further confirm the test result.

Recent studies have integrated sample-to-answer processes into a single biosensor to detect nucleic acids of pathogens and human antibodies against the pathogens, which could be further explored to detect COVID-19 infections. In addition, current works have also attempted to improve detection sensitivity, simplicity and performance of biosensors. Assay sensitivity can be enhanced through enzyme-based signal enhancement (He et al., 2011), sample concentration (Moghadam et al., 2015) or much simpler fluidic-control strategies (Choi et al., 2016b). Further, nucleic acid tests, in particular, that involves three key steps should be simplified and integrated into a single biosensor for POC use. The biosensors discussed in this review show immense potential to be developed into a self-contained platform for the detection of COVID-19 infections outside the laboratory, particularly in the remote settings or developing areas.

In the future, more studies should focus on simplifying user steps and incorporating all key steps into a single biosensor at minimal cost (Loo et al., 2019). The capability of storing all reagents on chip is vital to eliminate the requirement for large storage equipment (Deng and Jiang, 2019) whereas, the multiplexing capability of biosensors would increase throughput (Dincer et al., 2017). As compared to other detection approaches such as fluorescence and electrochemical detection approaches, colorimetric detection represents the most common approach mainly due to its simplicity and the ability to observe signal with the naked eyes. The biosensors that produce colorimetric signals enable visual detection without requiring extra tools such as UV lamp, which would be helpful for rapid on-site diagnosis (Chan et al., 2016; Yang et al., 2019). Quantification, which provides more accurate readout has been achieved using smartphone apps which could be performed by untrained users at POC settings. Future studies should further improve the function of smartphone and specific smartphone apps that enable on-site data analysis while allowing data storage to track patient health status (Roda et al., 2016; Wang et al., 2017; Liang et al., 2019; Xu et al., 2020). Additionally, incorporating portable power sources such as batteries into biosensors would significantly advance their functionality especially in rural areas where electricity supply is limited (Zarei, 2017b). In short, emerging POC biosensors with the aforementioned capabilities could rapidly identify the spread of COVID-19 and guide appropriate health care, playing a key role in managing the outbreak.

## Author Contributions

The author confirms being the sole contributor of this work and has approved it for publication.

## Conflict of Interest

The author declares that the research was conducted in the absence of any commercial or financial relationships that could be construed as a potential conflict of interest.
